# Acceptance of an Informational Antituberculosis Chatbot Among Korean Adults: Mixed Methods Research

**DOI:** 10.2196/26424

**Published:** 2021-11-09

**Authors:** Agnes Jihae Kim, Jisun Yang, Yihyun Jang, Joon Sang Baek

**Affiliations:** 1 National Rehabilitation Center Seoul Republic of Korea; 2 Department of Industrial Design Yonsei University Wonju Republic of Korea; 3 Department of Psychology Yonsei University Seoul Republic of Korea; 4 Department of Cognitive Science Yonsei University Seoul Republic of Korea; 5 Department of Human Environment and Design Yonsei University Seoul Republic of Korea

**Keywords:** tuberculosis, chatbot, technology acceptance model, mobile phone

## Abstract

**Background:**

Tuberculosis (TB) is a highly infectious disease. Negative perceptions and insufficient knowledge have made its eradication difficult. Recently, mobile health care interventions, such as an anti-TB chatbot developed by the research team, have emerged in support of TB eradication programs. However, before the anti-TB chatbot is deployed, it is important to understand the factors that predict its acceptance by the population.

**Objective:**

This study aims to explore the acceptance of an anti-TB chatbot that provides information about the disease and its treatment to people vulnerable to TB in South Korea. Thus, we are investigating the factors that predict technology acceptance through qualitative research based on the interviews of patients with TB and homeless facility personnel. We are then verifying the extended Technology Acceptance Model (TAM) and predicting the factors associated with the acceptance of the chatbot.

**Methods:**

In study 1, we conducted interviews with potential chatbot users to extract the factors that predict user acceptance and constructed a conceptual framework based on the TAM. In total, 16 interviews with patients with TB and one focus group interview with 10 experts on TB were conducted. In study 2, we conducted surveys of potential chatbot users to validate the extended TAM. Survey participants were recruited among late-stage patients in TB facilities and members of web-based communities sharing TB information. A total of 123 responses were collected.

**Results:**

The results indicate that perceived ease of use and social influence were significantly predictive of perceived usefulness (*P*=.04 and *P*<.001, respectively). Perceived usefulness was predictive of the attitude toward the chatbot (*P*<.001), whereas perceived ease of use (*P*=.88) was not. Behavioral intention was positively predicted by attitude toward the chatbot and facilitating conditions (*P*<.001 and *P*=.03, respectively). The research model explained 55.4% of the variance in the use of anti-TB chatbots. The moderating effect of TB history was found in the relationship between attitude toward the chatbot and behavioral intention (*P*=.01) and between facilitating conditions and behavioral intention (*P*=.02).

**Conclusions:**

This study can be used to inform future design of anti-TB chatbots and highlight the importance of services and the environment that empower people to use the technology.

## Introduction

### Background

Tuberculosis (TB) is a highly infectious disease and one of the top 10 causes of death worldwide, claiming approximately 4000 lives a day [1]. Each year, millions of people continue to fall ill with TB, a preventable and curable disease [2]. Among the member countries of the Organization for Economic Cooperation and Development, South Korea has the highest incidence of and mortality rates due to TB [2]. It remains a debilitating disease in the South Korean context in that the treatment is already generalized, but its prevalence and mortality rates are unevenly distributed among social classes [1,3]. Its eradication has been difficult owing to both stigmatization and insufficient understanding of the disease that cause delays in diagnosis and treatment [4]. Approximately one-quarter of the world’s population is estimated to be infected with TB, and approximately 5%-10% of those at risk of infection develop active TB in their lifetime [1,5].

### Mobile Health Interventions for TB Control

In recent years, mobile health (mHealth) has rapidly emerged as a vehicle for delivering better health services at a lower cost, regardless of time and place [6]. It is used to treat a wide range of infectious diseases, including TB. An extensive investigation on the use of digital technologies for TB control reports various mobile technologies applied for treatment adherence, program management, and e-learning related to TB [7]. These technologies include video-observed treatment (VOT), SMS text messages, mobile apps, voice calls, and mobile phone 3D-printed induration. mHealth apps assist medical staff with patient adherence monitoring (eg, apps for direct observed treatment [DOT] and VOT), dosage adjustment based on patient conditions, and provision of information about diagnosis and management of TB [7,8]. They inform patients and people vulnerable to TB about the disease and its therapy, provide diagnostics based on data input, and evaluate treatment costs. They are also used to trace people who have been exposed to the disease, monitor and track patients, and create laboratory reports [7]. The number of mHealth apps has more than doubled since 2016, evidencing the increasing demand for a new approach to TB control. It is also noteworthy that 39 out of the total 55 apps (71%) are only provided in English, thereby limiting access to non–English-speaking countries, where the highest prevalence of TB cases is observed [7].

### Chatbots

Chatbots are a conversational agent, a software program that interacts with natural language, and have emerged as a new form of mHealth service [9,10]. Chatbots are useful for providing information to users with low literacy: users interact with them through dialog, a universal form of interaction. Furthermore, they can provide information in formats that are accessible to people with low literacy, such as images, sounds, and videos [9]. Thus, they are relatively easy to learn and are also age-friendly. From the perspective of health care providers, chatbots can save time and labor [11], in addition to providing continuous treatment management plans, motivation for patients with chronic diseases, and access to real-time information [8,12].

However, despite the expansion of mHealth solutions for TB control and the potential of chatbots, little research has been conducted on applying these tools to the management of TB. To the best of our knowledge, few studies have attempted to develop chatbots and virtual agents to support information accessibility for patients with TB [8]. It is essential to understand the exact factors that predict the acceptance of chatbots by potential users before we introduce them to a TB eradication program, which indeed underscores that the success of digital interventions in health care will depend on how well users accept the technology [13]. Furthermore, understanding the factors that increase the use of chatbots would accelerate the acceptance of this technology among the people most at risk of contracting TB. For this reason, we developed an anti-TB chatbot to bridge the gap between technology and people and studied its acceptance based on the factors that predict potential users, using the Technology Acceptance Model (TAM).

### Context of Study: Anti-TB Chatbot

In 2019, we developed an anti-TB chatbot that provides information about the disease, its treatment, and TB hospitals and facilities. It targets people vulnerable to TB, as well as those affected by it. Textbox 1 presents the features of the chatbot.

**Antituberculosis chatbot feature summary.**

**Feature summary**

1. Providing information on tuberculosis
  - Functions
    Overview of tuberculosis
    Diagnosis of tuberculosis
    Tuberculosis treatment
    Information on drugs to treat tuberculosis
    Side effects of tuberculosis drugs
    Screening for contact and latent tuberculosis infection
2. Providing information on hospitals and facilities
  - Functions
    Institutions for tuberculosis screening and treatment
    Tuberculosis treatment support project
    Tuberculosis treatment support facility
    Information on welfare and administration related to tuberculosis
    Information on welfare facilities related to tuberculosis

The chatbot was built on an open-source platform and operates within an instant messenger app called Kakao Talk. An advantage of using this platform is that the medium through which users interact with the chatbot, that is, the messenger app, is widely used in South Korea, with over 72% of the total population or roughly 36.6 million people using it [14]. This makes the chatbot highly accessible as most people already have experience in using the app. The open-source platform builder uses machine learning to respond and adapt to diverse conversation patterns. This allows for the accuracy and relevance of the chatbot responses to improve as more user data accumulate.

The knowledge base was obtained from the information provided by the Korea Disease Control and Prevention Agency. We acquired the content with permission and then reorganized it in a dialog format. In addition to the text information, multimedia content was actively adopted, considering the tendency of low health literacy level of the poor and older people [15], who are characterized by a higher-than-average incidence of TB [16]. The curated content was examined by medical staff at a Seoul municipal hospital before publication. Gamification elements, including quizzes and prizes, were also adopted to motivate learners to engage with the chatbot [17].

We gave the chatbot the personality of a doctor. A chatbot with identity cues, such as a name, profile, and language style, is perceived as more empathetic, friendly, and personal [18,19]. Dr Colochman, the personality of the chatbot, is a retired doctor with a long record of treating patients with TB at a municipal hospital and is now working voluntarily for TB hospitals and support facilities. Its identity is conveyed through portraits, names, and intonation. Users encounter Dr Colochman for the first time during the tutorial that provides information on the chatbot and the instructions on how to use it in (what is supposedly) Dr Colochman’s voice.

**Figure 1:**
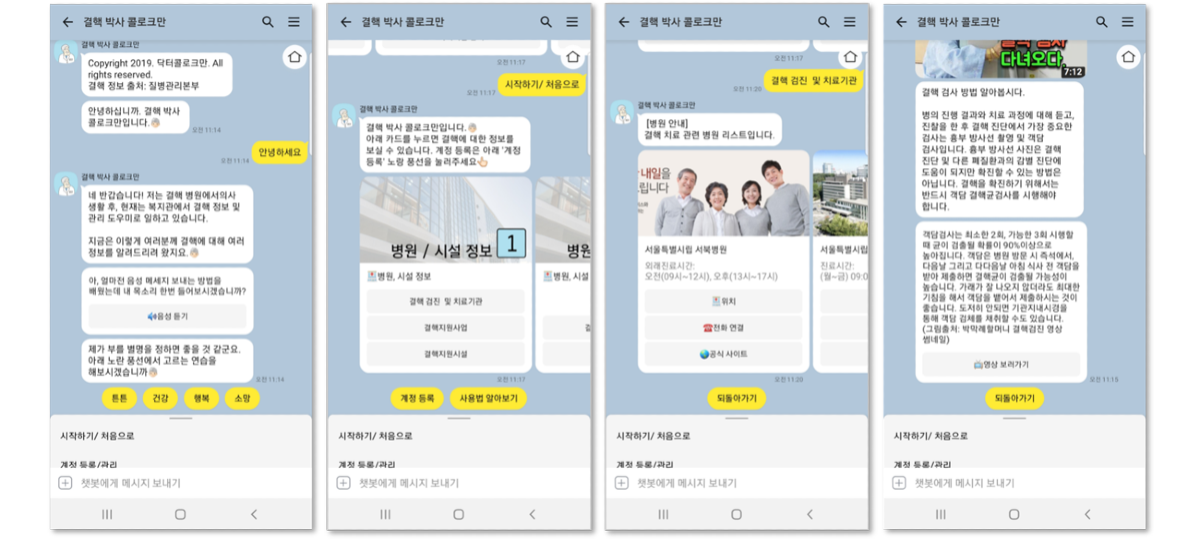
Antituberculosis chatbot user interface.

The chatbot provides graphic and text information on the disease, its treatment, and neighboring TB facilities. Users navigate the content by scrolling the page vertically and horizontally. They communicate with the chatbot by selecting menus at the bottom of the screen, pushing buttons, or typing texts (Figure 1).

### TAM and Chatbots

Davis et al [20] developed the TAM to investigate users’ intent to accept various technologies, including chatbots, and the factors that predict their decisions [20,21]. The key determinants used to study the acceptance of new technologies with the TAM are perceived usefulness, perceived ease of use, attitude, and behavioral intention. In this study, perceived usefulness is defined as the extent to which users think using anti-TB chatbots is helpful for TB management, and perceived ease of use is the extent to which users think using anti-TB chatbots is convenient and low-effort. According to the TAM, the adoption of a particular technology is governed by individual perceptions of usefulness and ease of use [21].

The TAM is widely used in technology acceptance research; however, it can predict only approximately 40% of the overall explanatory power [22]. A number of extended TAMs have been proposed to overcome the limitations of the original model. Venkatesh and Davis [23] developed the TAM2, adding social influence processes such as subjective norms, voluntariness, and image as external constructs of perceived usefulness. Social influence in our context is the extent to which users think that important others believe in using the anti-TB chatbot. Venkatesh and Davis [24] developed the Unified Theory of Acceptance and Use of Technology, which is the latest derivative of the TAM, adding facilitating conditions as a determinant of behavioral intention. Facilitating conditions here refer to the extent to which users think organizational and technical infrastructure exists to support the use of anti-TB chatbots.

Among the studies that have validated the TAM, some extended the model to address different contexts and populations, including the acceptance and continuous use of chatbots. For example, Huang and Chueh [25] reported that perceived accuracy and ease of use increased pet owners' satisfaction with veterinary consultation chatbots. Ashfaq et al [26] found that perceived enjoyment, usefulness, and ease of use are significant predictors of the continuance intention of chatbot-based customer service. In a study that investigated the acceptance of the health chatbot, Softić et al [27] identified the lack of users’ trust and qualified medical opinion as barriers; data confidentiality, speed of access to information, information security, and ease of use as facilitators; and reduced time spent on visiting doctors, increased access and care of patients, and enhanced protection of patient data as motivators for using a chatbot.

In the absence of studies that explain the acceptance of chatbots in the context of TB control, we aim to explore the benefits and concerns regarding accepting an anti-TB chatbot as perceived by potential users, to provide an extended TAM that can better predict the acceptance of anti-TB chatbots. Thus, we present studies 1 and 2. Study 1 aims to identify the factors that predict the acceptance of anti-TB chatbots through interviews with patients with TB and homeless facility personnel. On the basis of the interview results, we derived an operational definition of the questionnaire items and identified the factors for the extended TAM. Study 2 aims to verify the proposed theoretical model and identify the factors predicting the acceptance of an anti-TB chatbot.

## Methods

### Study 1

#### Data Collection

To collect data for study 1, we conducted interviews with potential users of our anti-TB chatbot. Interviewees were recruited by posting a notice at a municipal TB hospital. The participants were selected using convenience sampling among people who have or had TB. People who could neither understand nor respond to the questionnaire provided in Korean were excluded. In total, 16 patients with TB received a gift worth US $50. We also conducted a focus group interview with 10 experts on TB from the academia, hospitals, shelters, support facilities, and housing providers for homeless people who have worked for patients with TB and thus have sufficient knowledge about them and are willing to use the chatbot or introduce it to them. Participant information is presented in Table 1.

**Table 1:** Participant information of study 1 (N=26; site: Seoul; year: 2020).

Demographics				Values, n (%)
**Patients with TB^a^**				
	Gender			
		Male	16 (100)	
		Female	0 (0)	
	Age (years)			
		30s	2 (13)	
		40s	3 (19)	
		50s	3 (19)	
		60s	6 (38)	
		70s	2 (13)	
	Experience of smartphone use			
		Yes	9 (56)	
		No	7 (44)	
	Experience of chatbot use			
		Yes	0 (0)	
		No	16 (100)	
**Experts in treating TB**				
	Academia		1 (10)	
	Hospital		1 (10)	
	Shelters		2 (20)	
	Support facilities		5 (50)	
	Housing provider		1 (10)	

^a^TB: tuberculosis.

#### Procedure

Data collection followed the protocols of the American Psychological Association (APA) ethical principles and code of conduct [28]. However, institutional review board approval was not sought. The interviewees were presented with the aim of the study, its procedure and duration, anticipated benefits, and data protection policy. Written informed consent was obtained from those who agreed to participate in the research for recording texts, images, and voices. All the data were transcribed and pseudoanonymized. The interviews were conducted in the following order: (1) introduction to the research, (2) explanation of the data protection policy and collection of informed consent, (3) introduction to the chatbot and instructions on how to use it, (4) installation of the messenger app (if not already installed) and trial of the chatbot, and (5) interview session. All interviews were conducted in the Korean language.

#### Data Analysis

We analyzed the collected data using thematic analysis in ATLAS.ti, a qualitative data analysis and research tool. Three researchers designed the coding frame to analyze the interviewees' attitude or intent to accept the anti-TB chatbot. We classified the results into a set of subthemes, which were clustered into the main themes. These main themes were assigned as TAM factors.

### Study 2

#### Hypotheses

Study 2 aimed to evaluate factors that predict the acceptance of the anti-TB chatbot. According to Davis et al [20], perceived usefulness and perceived ease of use were the primary factors that predicted the attitude toward a new technology under the TAM. Moreover, perceived ease of use was associated with perceived usefulness. Finally, the attitude toward the technology determined the behavioral intention [23]. Thus, we proposed the following hypotheses:

*Hypothesis 1:* Attitude toward the chatbot would be positively predicted by perceived usefulness.

*Hypothesis 2:* Attitude toward the chatbot would be positively predicted by perceived ease of use.

*Hypothesis 3:* Perceived usefulness would be positively predicted by perceived ease of use.

*Hypothesis 4:* Behavioral intention would be positively predicted by attitude toward the chatbot.

**Figure 2:**
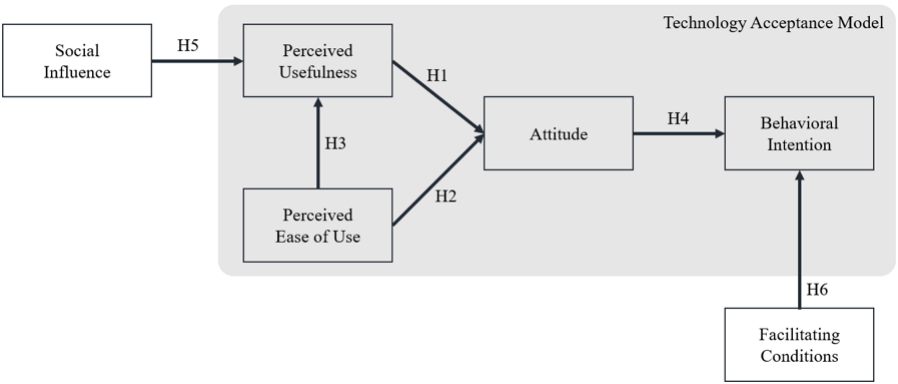
Hypothetical model of study 2.

Study 1 demonstrated that social influence and facilitating conditions were relevant to the acceptance of the chatbot by patients with TB. In previous studies that modeled technology acceptance, social influence such as subjective norm, voluntariness, and image is known as an external construct of perceived usefulness [23], while facilitating conditions such as internal and external resources are determinants of behavioral intention [24]. We thus built additional hypotheses as follows and conducted a study that considered them as variables in the research model (Figure 2):

*Hypothesis 5:* Perceived usefulness would be positively predicted by social influence.

*Hypothesis 6:* Behavioral intention would be positively predicted by facilitating conditions.

#### Data Collection

We conducted both offline and web-based surveys, considering that older adults and other vulnerable groups have limited access to the internet. In the offline survey, we recruited participants at TB facilities that were mainly used by patients in the late stage of TB treatment, who can take medication on their own after discharge from the hospital. The research team visited the facility and instructed and provided assistance to those who expressed their willingness to participate. In the web-based survey, participants were recruited from web-based communities that share information on TB. The web-based survey was distributed among the potential users of the anti-TB chatbot, and their responses were collected via Google Forms. All participants received a monetary reward worth US $5.

#### Procedure

As in study 1, the data collection process in study 2 was guided by the protocols of the APA ethical principles and code of conduct [28]. All survey participants were asked to read (or were told, if they could not read) the introduction page of the survey describing the purpose of anti-TB chatbot use, its procedure and duration, anticipated benefits, and the data protection policy. Written informed consent was obtained from those who agreed to participate in the research for collecting texts. We then introduced the main screen and dialogs of the app, which informed the participants of the character and functionality of the chatbot. All the data were pseudoanonymized.

#### Questionnaire Development

The questionnaire was developed based on the theoretical framework of the TAM and the findings from study 1. It consisted of 32 items inquiring about demographic and attitudinal data—participants were asked general questions on demography and experience with chatbots and specific questions regarding their attitude toward the anti-TB chatbot. The attitudinal components were measured using a 7-point Likert-type scale, where the choice of answers ranged from *strongly disagree* (score=1) to *strongly agree* (score=7). The language used in the questionnaire was revised to consider the context of TB and reflect the digital literacy of potential users, as inferred from the results of study 1. The details of the questionnaire items for each construct are presented in Multimedia Appendix 1.

#### Data Analysis

A total of 127 cases were collected in March 2020. After the screening, 4 cases were excluded: there were missing values in 3 cases, and a straight line was found in 1 case. We used the partial least squares structural equation modeling (PLS-SEM) approach to statistically analyze and process the collected data using SmartPLS 3.0, a dedicated structural equation program with a strong verification power, even for small sample sizes. First, we used the PLS algorithm to evaluate the measurement model. This was followed by bootstrapping and blindfolding techniques for evaluation and hypothesis testing of the structural models.

## Results

### Study 1

#### Perceived Usefulness

**Figure 3:**
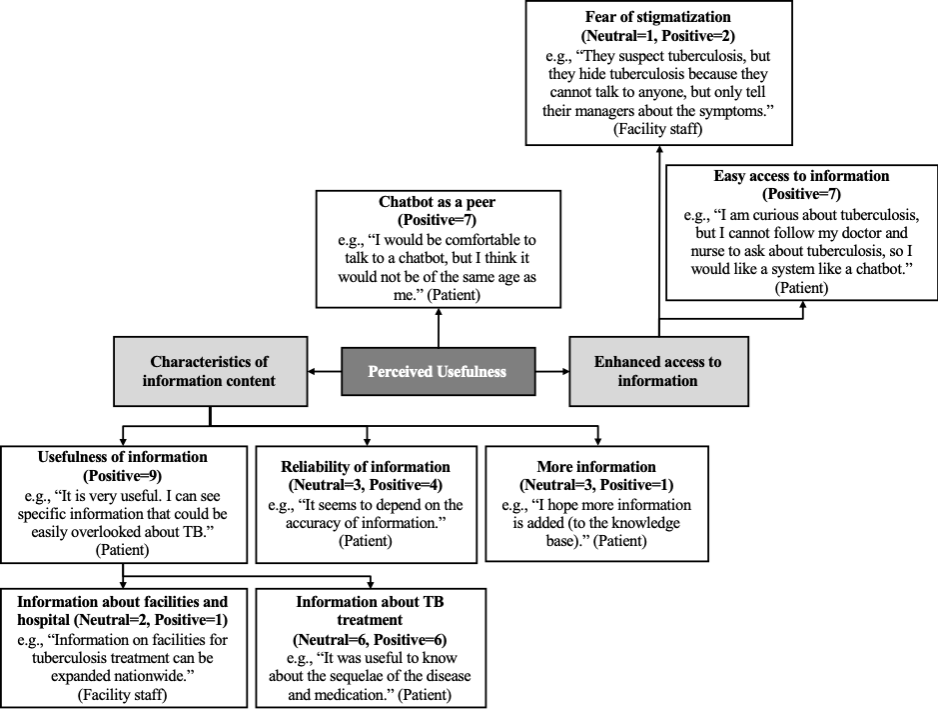
Perceived usefulness of the antituberculosis chatbot among potential users (n=the number of times a theme was mentioned by the interviewees of study 1).

The interviewees noted that the usefulness of the chatbot was associated with the characteristics of the information content, the chatbot's ability to communicate in a similar manner as a peer, and enhanced access to information (Figure 3). In terms of information content, they expected not only useful and reliable information about TB and its treatment but also more content. For example, they considered the fact that the chatbot currently provides information on facilities for TB treatment in Seoul only, which is a limitation. They found information on TB treatment, including hospitals and support facilities for patients with TB, to be most useful. Finally, they anticipated that the chatbot could help reduce the workload of medical staff while increasing patients’ access to the necessary information and reducing the risk of stigmatization.

#### Perceived Ease of Use

**Figure 4:**
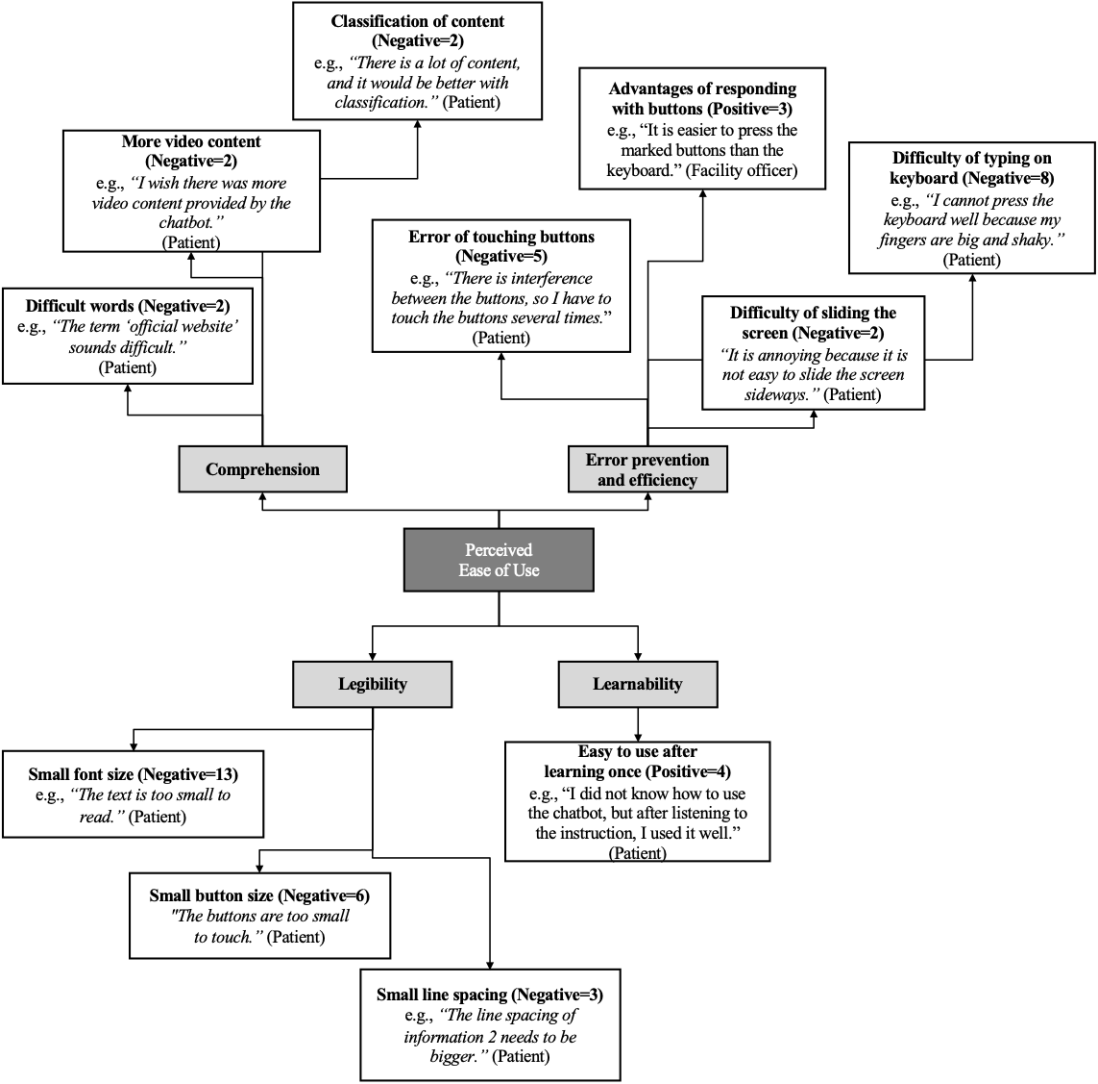
Perceived ease of use of the antituberculosis chatbot among potential users (n=the number of times a theme was mentioned by the interviewees of study 1; positive and neutral comments in normal and negative comments in italics).

Regarding the perceived ease of use of the anti-TB chatbot, the interviewees mentioned the following themes: legibility, comprehension, error prevention and efficiency, and learnability (Figure 4). Legibility is defined as the ability “to see, distinguish, and recognize the characters and words in a text” and is influenced by visual design [29]. In the anti-TB chatbot, legibility issues included inadequate font and button sizes and narrow line spacings. Comprehension *measures whether a user can understand the intended meaning of a text and can draw the correct conclusions from the text* [29]. Related issues included difficult wording, audio-visual information, and unorganized information. Error prevention and efficiency were often related to usability functions supported by the chatbot development platform. The open-builder platform we used provided a simple but functionally constrained environment to develop the chatbot. For example, users can respond to a question from the chatbot either by touching a button or by typing on a virtual keyboard. Interviewees found it difficult to type answers due to the small button size and distance between them, which however could not be adjusted on the platform. The interviewees mentioned that horizontally navigating the information by sliding the screen sideways was troublesome. In terms of learnability, the interviewees quickly learned how to navigate the chatbot after they were given proper instructions.

#### Facilitating Conditions

**Figure 5:**
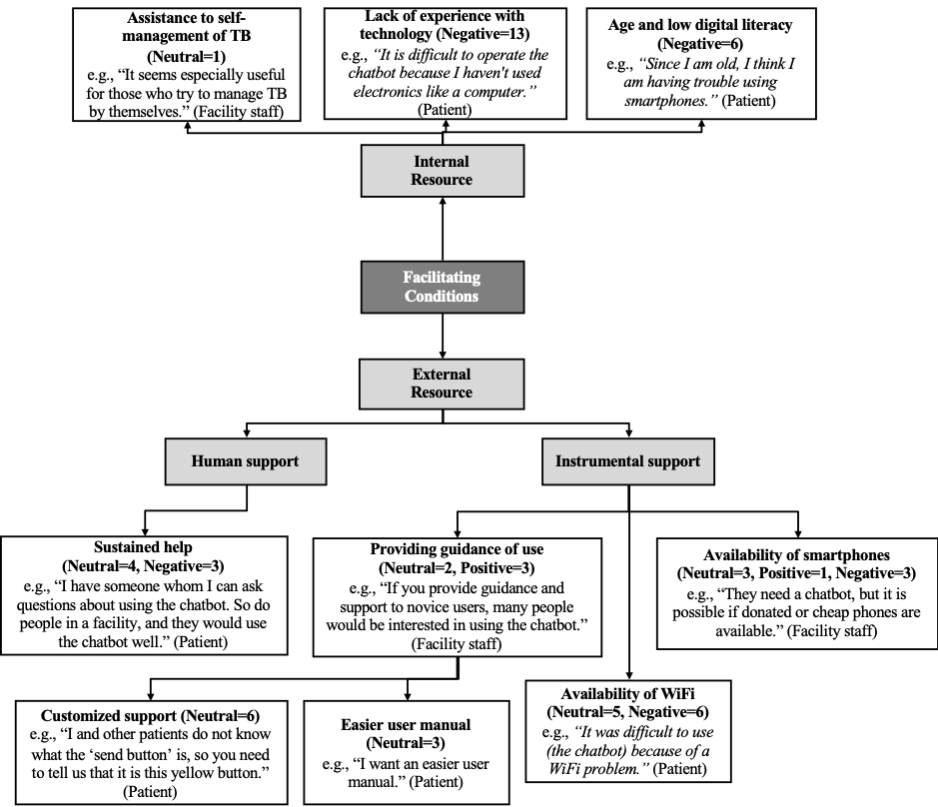
Facilitating conditions of the antituberculosis chatbot (n=the number of times a theme was mentioned by the interviewees of study 1; positive and neutral comments in normal and negative comments in italics).

Facilitating conditions associated with the interviewees’ acceptance of the anti-TB chatbot were classified as internal and external resources (Figure 5). The former included the user's will to self-manage the disease, their experience of using smartphones and computers, and their age. For example, older interviewees who had no experience of using a smartphone hesitated to use the chatbot. External resource was further subdivided into instrumental and human supports. The former included the availability of instructions and guidance on how to use the chatbot, availability of a smartphone or computer, and access to the internet. Several interviewees lacked basic digital literacy skills and required explanations for simple tasks such as touching the *send message* button. This further suggests the need for an easy-to-understand user manual. The interviewees’ attitude toward the acceptance of the chatbot differed depending on the availability of human support, that is, someone who could help them use the chatbot effectively.

#### Social Influence

**Figure 6:**
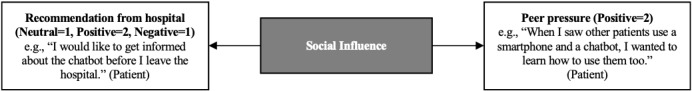
Social influence of the antituberculosis chatbot in potential users. It should be noted that n=number of times a theme was mentioned by the interviewees of study 1.

The social influence on the use of the anti-TB chatbot was governed by recommendations from professionals treating TB and the context of use. Interviewees mentioned that recommendations from hospitals would facilitate their adoption of the chatbot (Figure 6). The fact that their peers used the chatbot would also motivate them to accept the new technology.

### Study 2

#### Demographics

Participants’ ages ranged from 22 to 85 years, with almost equal participation by men and women. Most respondents did not have any history of TB, and approximately half had no experience using chatbots. Out of 123 participants, 120 (97.5%) participants had already used the messenger app (Table 2).

**Table 2:** Participant demographics of study 2 (N=123; site: Seoul; year: 2020).

Demographics			Values, n (%)
**Gender**			
	Female	60 (48.7)	
	Male	63 (51.2)	
**Age (years)**			
	22 to 29	26 (21.1)	
	30 to 39	33 (26.8)	
	40 to 49	34 (27.6)	
	50 to 59	9 (7.3)	
	60 to 85	21 (17.1)	
**History of tuberculosis**			
	Yes	16 (13)	
	No	107 (86.9)	
**Experience of using the messenger app**			
	Yes	120 (97.5)	
	No	3 (2.5)	
**Chatbot experience**			
	Yes	61 (49.5)	
	No	62 (50.4)	

#### Evaluation of the Measurement Model

The measurement models of study 2 using PLS-SEM were evaluated for internal reliability, convergent validity, and discriminant validity. The internal reliability was assessed using Cronbach α and composite reliability, in which a value greater than .70 for each indicates acceptable internal consistency [30]. To assess the convergent validity, the average variance extracted (AVE) was used, with a recommended value of 0.50 [31]. The results are presented in Table 3. Cronbach α ranged from .798 to .932, and the composite reliability ranged from 0.868 to 0.951, indicating strong internal reliability. Table 3 also presents the estimated construct loading for the study, which ranged from 0.801 to 0.941, and the AVE, which ranged from 0.625 to 0.831, which are greater than the corresponding recommended levels. Therefore, the conditions for convergent validity were satisfied in this study.

**Table 3:** Reliability and convergent validity of the measurement model in study 2.

Construct and items		Factor loadings	Cronbach α		Composite reliability coefficient		Average variance extracted	
**PU^a^**			.854		0.902		0.697	
	PU 1	0.893						
	PU 2	0.859						
	PU 3	0.860						
	PU 4	0.783						
**PEOU^b^**				.927		0.948		0.822
	PEOU 1	0.870						
	PEOU 2	0.892						
	PEOU 3	0.941						
	PEOU 4	0.920						
**SI^c^**			.858		0.904		0.702	
	SI 1	0.801						
	SI 2	0.852						
	SI 3	0.887						
	SI 4	0.808						
**FC^d^**			.798		0.868		0.625	
	PR 1	0.860						
	PR 2	0.852						
	PR 3	0.635						
	PR 4	0.847						
**ATC^e^**			.899		0.930		0.768	
	ATC 1	0.834						
	ATC 2	0.893						
	ATC 3	0.899						
	ATC 4	0.878						
**BI^f^**			.932		0.951		0.831	
	BI 1	0.851						
	BI 2	0.931						
	BI 3	0.924						
	BI 4	0.937						

^a^PU: perceived usefulness.

^b^PEOU: perceived ease of use.

^c^SI: social influence.

^d^PR: facilitating conditions.

^e^ATC: attitude to chatbot.

^f^BI: behavioral intention.

Discriminant validity was assessed using the square root of the AVE in the cross-loading matrix. To establish a satisfactory discriminant validity of the model, the square root of the AVE for a given construct should be greater than its correlation with other constructs [31]. This, in turn, implies that the diagonal elements must be larger than the entries in the corresponding columns and rows of the matrix. The results shown in Table 4 reveal that all the constructs in this study confirm the discriminant validity of the data.

**Table 4:** Discriminant validity of the measurement model in study 2.

Constructs	Perceived usefulness	Perceived ease of use	Social influence	Facilitating conditions	Attitude to chatbot	Behavioral intention
Perceived usefulness	0.835	0.512	0.81	0.519	0.714	0.588
Perceived ease of use	0.512	0.906	0.422	0.707	0.325	0.410
Social influence	0.81	0.422	0.838	0.508	0.743	0.664
Facilitating conditions	0.519	0.707	0.508	0.791	0.421	0.494
Attitude to chatbot	0.714	0.325	0.743	0.421	0.876	0.713
Behavioral intention	0.588	0.410	0.664	0.494	0.713	0.911

#### The Structural Model

**Figure 7:**
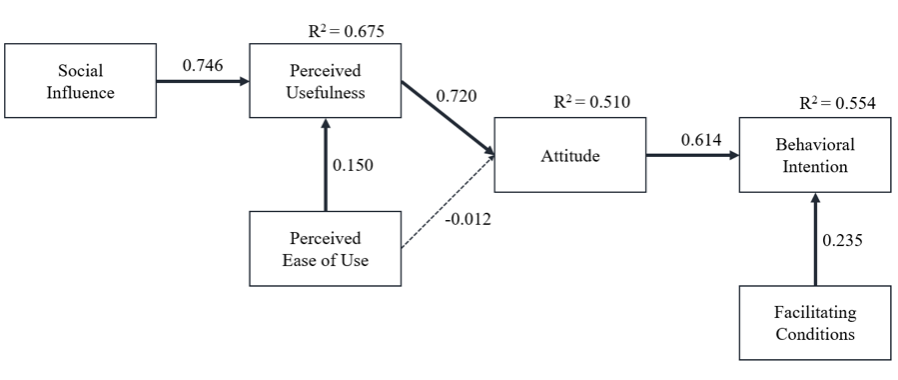
Path analysis results for study 2.

The results of the structural model for the TAM are shown in Figure 7 and Table 5. The significance of the path coefficients was assessed using bootstrapping with 5000 samples. The results indicate that attitude toward the chatbot was positively predicted by perceived usefulness (hypothesis 1 supported; *P*<.001) but was not significantly predicted by perceived ease of use (hypothesis 2 not supported; *P*=.88). Perceived usefulness was positively predicted by perceived ease of use (hypothesis 3 supported; *P*<.001) and social influence (hypothesis 5 supported; *P*<.001). Social influence and perceived ease of use explained 67.5% of the variance in perceived usefulness. Finally, behavioral intention was positively predicted by attitude toward the chatbot (hypothesis 4 supported; *P*<.001) and facilitating conditions (hypothesis 6 supported; *P*=.03). Overall, attitude toward the chatbot and facilitating conditions explained 55.4% of the variance in behavioral intention.

**Table 5:** Results of the structural model in study 2.

Endogenous variable and exogenous variable		β	*t* value	*P* value	
**Perceived usefulness**					
	Perceived ease of use	.15	2.062	.04	
	Social influence	.746	12.023	<.001	
**Attitude to chatbot**					
	Perceived usefulness	.720	11.314	<.001	
	Perceived ease of use	−.012	0.151	.88	
**Behavioral intention**					
	Facilitating conditions	.235	2.242	.03	
	Attitude to chatbot	.614	7.438	<.001	

#### Multigroup Analysis

We also performed a PLS multigroup analysis (PLS-MGA) by dividing the participants into 2 groups based on their history of TB. There were 107 participants with a history of TB and 16 with no history of TB. The results indicated that perceived usefulness was positively predicted by social influence in both groups (Table 6). Facilitating conditions were predictive of behavioral intention in the TB history group, whereas the attitude toward the chatbot was predictive of behavioral intention in the non–TB history group.

**Table 6:** Results of the multigroup analysis.

Path	TB^a^ history group	Non–TB history group	Difference	TB history group	Non–TB history group	*P* value
	β	β	β	*P* value	*P* value	
PU^b^→ATC^c^	.662	.733	−.071	.002	<.001	.72
PEOU^d^→ATC	.186	−.046	.233	.41	.60	.34
PEOU→PU	−.118	.194	−.313	.56	.008	.13
ATC→BI^e^	.113	.66	−.547	.66	<.001	.01
SI^f^→PU	.906	.726	.18	<.001	<.001	.34
FC^g^→BI	.826	.175	.651	.002	.07	.02

^a^TB: tuberculosis.

^b^PU: perceived usefulness.

^c^ATC: attitude to chatbot.

^d^PEOU: perceived ease of use.

^e^BI: behavioral intention.

^f^SI: social influence.

^g^FC: facilitating conditions.

## Discussion

### Principal Findings

This study aimed to propose a chatbot that provides information for the prevention and treatment of TB and identify factors that predict the acceptance of the chatbot. We conducted interviews with 16 patients with TB and 10 experts in TB and identified the factors that predict the acceptance of the anti-TB chatbot in study 1. From the results, we found social influence and facilitating conditions as additional factors in the extended TAM model. In study 2, we proposed an extended TAM model capable of predicting the acceptance of the anti-TB chatbot and evaluated it. We found that social influence was a strong predictor of perceived usefulness, regardless of history of TB. Study 1 suggests that social influence can arise from both health care experts and peers. Regarding users' behavioral intention, the predictive factor varied in the participants’ history of TB. Overall, our findings were consistent with those of other researchers [18,19,21], indicating that (1) perceived usefulness was predicted by social influence, (2) attitude was predicted by perceived usefulness, and (3) attitude toward the system and facilitating conditions predicted behavioral intention.

### Perceived Usefulness

Our study confirmed that people needed information about the disease, as well as TB hospitals and support facilities. It also suggested that the reliability of the information provided by the chatbot is crucial to perceived usefulness and eventually the acceptance of the chatbot. Although this may sound rather obvious, existing mHealth apps that provide information on TB have been found to contain errors such as spelling and grammatical mistakes, outdated information, and wrong and potentially harmful content, according to a recent study that investigated 29 e-learning and information apps on TB [7]. The reliability of the information can be achieved by using trusted sources, having the content examined by experts before publication, and keeping it up to date through continuous maintenance.

Perceived usefulness was significantly predictive of people’s attitude toward the anti-TB chatbot if they have experienced TB. When people seek information about TB, stigmatization and its consequences (eg, social isolation and reduced economic opportunities) can be barriers to active information seeking and timely access to necessary services [32]. We expect that the anti-TB chatbot can contribute to lowering this barrier by facilitating access to information and reducing the risk of stigmatization (see Figure 4 for a glimpse of the chatbot experience). For patients with TB, the primary channel through which they receive information related to the disease is the medical staff. However, due to limited time at hand, medical staff provide selective information. The anti-TB chatbot can reduce staff workload while providing patients with the necessary information when needed. In other words, it bridges the distance between patients and medical staff by acting as a virtual assistant [33]. It also mitigates the information asymmetry between the 2 parties by empowering patients with the ability to access the information they need.

### Perceived Ease of Use

Study 2 confirmed that perceived ease of use was predictive of perceived usefulness but not predictive of the attitude toward technology. The latter result has been observed in studies where participants were proficient in using the technology (eg, responses of experienced mobile phone users to a mobile app or a chatbot) [34,35]. The same trend was observed in study 2, where there was a roughly even distribution in age of the survey participants (22 to 85 years). Does this imply that participants of different ages, and possibly varied levels of digital literacy, were proficient in using the chatbot that they were introduced for the first time? If so, what aspects of the chatbot are associated with proficiency? We speculate that this may be due to the popularity of the platform on which the anti-TB chatbot runs, that is, the messenger app widely used by people. The familiar user interface of the chatbot may have been transferred to the perceived proficiency in the use of the chatbot and a positive opinion of its utility. Thus, we conclude that the perceived usefulness of a chatbot can increase when its user interface is familiar to the target users.

### Social Influence

In study 1, we observed social influence acting on the interviewees when a staff member in the hospital or TB treatment facility recommended the use of the anti-TB chatbot or when a peer introduced them. Thus, social influence can have a positive impact on the perceived usefulness of the chatbot. In a study that investigated the acceptance of conversational agents for disease diagnosis, social influence was identified as a factor influencing users’ intention to adopt or use a chatbot [9]. It has also been reported that users’ trust in providers and chatbots predicts performance expectancy. Performance expectancy refers to the degree to which using a chatbot will provide benefits to users in improving their health conditions [24].

Social influence can be derived from the authority and credibility of the service provider (ie, hospital) and those who have (expert or user) knowledge about the disease and technology. Among the different types of social influence was the peer pressure from other people who use a smartphone and a chatbot. For example, a patient with TB whom we met in study 1 was among several patients who did not have a smartphone and were eager to learn to use the smartphone and the anti-TB chatbot (Figure 6). Considering that there is still a large population who cannot access mHealth solutions, our findings reiterate a barrier to these technologies and simultaneously a strong demand for them that remains to be met. It is beyond the scope of this study to discuss how to meet this demand, but we introduce some of the existing efforts and emphasize the need for facilitating conditions in the Facilitation Condition section.

### Facilitating Condition

Facilitating condition is strongly associated with the acceptance of chatbots by patients with TB and thus should be considered when designing an anti-TB chatbot. TB occurs more commonly in older adults and low-income groups. It is also these groups who find it most challenging to access and use mHealth solutions. They are often reluctant to accept new solutions, such as chatbots, due to a lack of internal resources (eg, information on and capabilities to use mHealth solutions) according to study 1. However, this lack can be compensated by the provision of external support, such as a peer who can help them learn how to use a chatbot or smartphone tutorials. Existing chatbot-related studies tend to focus on the efficiency and usefulness of these technologies [11,36-38]. However, our findings suggest that it is equally important to design facilitating conditions from the perspective of users to encourage and accelerate their acceptance of chatbots. In other words, both services and an environment that empowers people to use the chatbots should be designed to eradicate TB.

Trans-sectoral efforts have been made to disseminate smartphones among homeless people as a strategy to reinforce self-sufficiency and mitigate poverty. Organizations, such as the Community Technology Alliance, Seoul Municipality, and Underheard in New York, have implemented smartphone giveaway projects in which donated smartphones were delivered to homeless people and used to find accommodation, economic opportunities, and fulfill other basic needs [39,40]. These examples demonstrate the possibility of making mHealth solutions accessible to the bottom of the pyramid, although they do not report any integration with mHealth. On the basis of our empirical study, we cautiously argue that there is sufficient demand for mHealth solutions, including the anti-TB chatbot, among poor and older people. The question is how to deliver them in a scaled-up and sustained manner. As we witness the rapid growth of the mHealth industry and anticipate a variety of solutions for TB control, including chatbots, the facilitating conditions are all the more important for the democratization of these technologies, that is, the development of technologies for people who are most affected by TB should be concurrent with sustained efforts to empower them.

### Limitations

This study has several limitations. First, our hypotheses were evaluated using correlation methods; therefore, the derived model did not explain causal relationships among the identified constructs. Second, this study was conducted using a convenience sample, which limits the generalizability of our findings. Thus, future studies should conduct a more comprehensive inspection of how these individual differences are associated with the acceptance of technology using representative and larger samples [41]. With larger samples, we may also be able to identify additional external factors that are predictive of the acceptance of the anti-TB chatbot. Potential candidates include social support and stigma, which have been identified as relevant for treating TB [42-44]. Third, our sample with a history of TB is relatively small and homogeneous due to the invisibility caused by the fear of stigmatization. Although this study showed that the predictor of anti-TB chatbot acceptance depends on history of TB, the number of patients was not sufficient to obtain results with greater statistical and conceptual strengths. Finally, the impact of this study remains limited in the current environment, where technological advances are not accessible to many homeless people who can benefit from them. At the same time, we are reminded that technology alone cannot solve complex societal problems. We also need to invest in scaling up the ongoing efforts to empower these people (eg, digital literacy education) and build the necessary infrastructure (eg, provide mobile devices and services that they can afford and expand public Wi-Fi zones in low-income residential areas).

### Conclusions

Despite the expansion of mHealth solutions for TB control and the potential of chatbots to save costs and reduce the risk of stigma associated with the diagnosis and treatment of TB, few studies have sought to investigate the determinants of their adoption. In this context, we conducted 2 studies to develop an extended TAM that incorporates additional variables obtained from an empirical study with patients with TB and explain the intention to use a chatbot for TB control. The results showed that the intention to use the anti-TB chatbot was predicted by attitude toward the chatbot and facilitating conditions. Attitude toward the chatbot was positively predicted by its perceived usefulness but was not significantly predicted by perceived ease of use. The results also suggested that the perceived usefulness of the anti-TB chatbot was positively predicted by perceived ease of use and social influence. The importance of this study is to identify the underlying factors associated with the intention to use an anti-TB chatbot. These findings can be used to inform future design of anti-TB chatbots. For future work, it will be necessary to integrate the proposed model with other theories and factors that can help explain greater acceptance.

## Appendix

Multimedia Appendix 1 Definition for the constructs used in study 2.

## Abbreviations

AVE: average variance extracted
DOT: direct observed treatment
mHealth: mobile health
PLS-MGA: partial least squares multigroup analysis
PLS-SEM: partial least squares structural equation modeling
TAM: Technology Acceptance Model
TB: tuberculosis
VOT: video-observed treatment

## References

[1] World Health Organization (2013). Global Tuberculosis Report 2013.

[2] (2020). Global Tuberculosis Report 2020. World Health Organization.

[3] Christof C, Nußbaumer-Streit B, Gartlehner G (2020). WHO guidelines on tuberculosis infection prevention and control. Gesundheitswesen.

[4] Gebremariam MK, Bjune GA, Frich JC (2010). Barriers and facilitators of adherence to TB treatment in patients on concomitant TB and HIV treatment: a qualitative study. BMC Public Health.

[5] World Health Organization (2018). Latent Tuberculosis Infection: Updated and Consolidated Guidelines for Programmatic Management.

[6] Samhan B, Joshi K (2015). Resistance of healthcare information technologies; literature review, analysis, and gaps. Proceedings of the 48th Hawaii International Conference on System Sciences.

[7] Keutzer L, Wicha SG, Simonsson US (2020). Mobile health apps for improvement of tuberculosis treatment: descriptive review. JMIR Mhealth Uhealth.

[8] Iribarren SJ, Wallingford J, Schnall R, Demiris G (2020). Converting and expanding mobile support tools for tuberculosis treatment support: Design recommendations from domain and design experts. J Biomed Inform.

[9] Laumer S, Maier C, Gubler F (2019). Chatbot acceptance in healthcare: explaining user adoption of conversational agents for disease diagnosis. Proceedings of the 2019 European Conference on Information Systems.

[10] Laranjo L, Dunn A, Tong H, Kocaballi A, Chen J, Bashir R, Surian D, Gallego B, Magrabi F, Lau AY, Coiera E (2018). Conversational agents in healthcare: a systematic review. J Am Med Inform Assoc.

[11] Hoermann S, McCabe KL, Milne DN, Calvo RA (2017). Application of synchronous text-based dialogue systems in mental health interventions: systematic review. J Med Internet Res.

[12] Chaix B, Bibault J, Pienkowski A, Delamon G, Guillemassé A, Nectoux P, Brouard B (2019). When chatbots meet patients: one-year prospective study of conversations between patients with breast cancer and a chatbot. JMIR Cancer.

[13] Ngwatu BK, Nsengiyumva NP, Oxlade O, Mappin-Kasirer B, Nguyen NL, Jaramillo E, Falzon D, Schwartzman K, Collaborative Group on the Impact of Digital Technologies on TB (2018). The impact of digital health technologies on tuberculosis treatment: a systematic review. Eur Respir J.

[14] Piao M, Ryu H, Lee H, Kim J (2020). Use of the healthy lifestyle coaching chatbot app to promote stair-climbing habits among office workers: exploratory randomized controlled trial. JMIR Mhealth Uhealth.

[15] Sudore R, Mehta K, Simonsick E, Harris T, Newman A, Satterfield S, Rosano C, Rooks RN, Rubin SM, Ayonayon HN, Yaffe K (2006). Limited literacy in older people and disparities in health and healthcare access. J Am Geriatr Soc.

[16] (2020). Annual Report on the Notified Tuberculosis in Korea. Korea Centers for Disease Control and Prevention.

[17] Glover I (2013). Play as you learn: gamification as a technique for motivating learners. Sheffield Hallam University Research Archive (SHURA).

[18] Araujo T (2018). Living up to the chatbot hype: The influence of anthropomorphic design cues and communicative agency framing on conversational agent and company perceptions. Comput Hum Behav.

[19] Feine J, Gnewuch U, Morana S, Maedche A (2019). A taxonomy of social cues for conversational agents. Int J Hum Comput Stud.

[20] Davis FD, Bagozzi RP, Warshaw PR (1989). User acceptance of computer technology: a comparison of two theoretical models. Manag Sci.

[21] Venkatesh V (2000). Determinants of perceived ease of use: integrating control, intrinsic motivation, and emotion into the technology acceptance model. Inform Sys Res.

[22] Legris P, Ingham J, Collerette P (2003). Why do people use information technology? A critical review of the technology acceptance model. Inform Manag.

[23] Venkatesh V, Davis FD (2000). A theoretical extension of the technology acceptance model: four longitudinal field studies. Manag Sci.

[24] Venkatesh V, Morris MG, Davis GB, Davis FD (2003). User acceptance of information technology: toward a unified view. MIS Q.

[25] Huang D, Chueh H (2020). An analysis of use intention of pet disease consultation chatbot. Proceedings of the 4th International Conference on E-Society, E-Education and E-Technology.

[26] Ashfaq M, Yun J, Yu S, Loureiro SM (2020). I, Chatbot: Modeling the determinants of users’ satisfaction and continuance intention of AI-powered service agents. Telemat Informat.

[27] Softić A, Husić J, Softić A, Baraković S (2021). Health chatbot: design, implementation, acceptance and usage motivation. Proceedings of the 20th International Symposium INFOTEH-JAHORINA (INFOTEH).

[28] Psychological Association (2002). Ethical principles of psychologists and code of conduct. Am Psychol.

[29] Legibility, readability, and comprehension: making users read your words. Nielsen Norman Group.

[30] Hair JF, Black WC, Babin BJ, Anderson RE (2009). Multivariate Data Analysis.

[31] Hair JJ, Hult G, Ringle C, Sarstedt M (2016). A Primer on Partial Least Squares Structural Equation Modeling (PLS-SEM).

[32] Courtwright A, Turner AN (2010). Tuberculosis and stigmatization: pathways and interventions. Public Health Rep.

[33] Fadhil A (2018). arXiv.org.

[34] Yuan S, Ma W, Kanthawala S, Peng W (2015). Keep using my health apps: discover users' perception of health and fitness apps with the UTAUT2 Model. Telemed J E Health.

[35] Zarouali B, Van den Broeck E, Walrave M, Poels K (2018). Predicting consumer responses to a chatbot on Facebook. Cyberpsychol Behav Soc Netw.

[36] Kim S, Eun J, Oh C, Suh B, Lee J (2020). Bot in the bunch: facilitating group chat discussion by improving efficiency and participation with a chatbot. Proceedings of the 2020 CHI Conference on Human Factors in Computing Systems.

[37] Kong B, Wang E, Li Z, Lu W (2019). Study on the feature of electromagnetic radiation under coal oxidation and temperature rise based on multifractal theory. Fractals.

[38] Zamora J (2017). I'm sorry, dave, I'm afraid I can't do that: chatbot perception and expectations. Proceedings of the 5th International Conference on Human Agent Interaction.

[39] (2009). Hear the voices from the underheard. Underheard in New York.

[40] (2019). Community Technology Alliance.

[41] Lamela D, Figueiredo B, Morais A, Matos P, Jongenelen I (2020). Are measures of marital satisfaction valid for women with depressive symptoms? The examination of factor structure and measurement invariance of the Couple Satisfaction Index-4 across depression levels in Portuguese women. Clin Psychol Psychother.

[42] Milligan H, Iribarren SJ, Chirico C, Telles H, Schnall R (2021). Insights from participant engagement with the tuberculosis treatment support tools intervention: Thematic analysis of interactive messages to guide refinement to better meet end user needs. Int J Med Inform.

[43] Paz-Soldán Valerie A, Alban RE, Jones CD, Oberhelman RA (2013). The provision of and need for social support among adult and pediatric patients with tuberculosis in Lima, Peru: a qualitative study. BMC Health Serv Res.

[44] Suliman Q, Lim P, Said S, Tan K, Zulkefli N (2021). Survival analysis of time to early tb treatment interruption: a longitudinal study on psycho-social risk factors among newly diagnosed tb patients in a state of Malaysia. Res Square.

