# Supplementary material for: Acceptance of an Informational Antituberculosis Chatbot Among Korean Adults: Mixed Methods Research
Source: JMIR Mhealth Uhealth. 2021 Nov 9;9(11):e26424. doi: 10.2196/26424 (PMC8663686; doi:10.2196/26424)
Supplement: Multimedia Appendix 1 [file mhealth_v9i11e26424_app1.docx]

## Appendix

1. Definition for the constructs used in study 2

| Constructs | Items | Questions |
| --- | --- | --- |
| Perceived Usefulness  (PU) | PU 1 | Chatbot allows you to quickly find the information you want to know. |
|  | PU 2 | You will be able to find out the answer to your questions more easily through the chatbot. |
|  | PU 3 | Chatbot will provide accurate information. |
|  | PU 4 | Tuberculosis information can be obtained through chatbot without asking medical staff. |
| Perceived Ease of Use (PEOU) | PEOU 1 | I can easily learn how to use a chatbot. |
|  | PEOU 2 | Chatbot is easy to see. |
|  | PEOU 3 | Chatbot seems easy to use. |
|  | PEOU 4 | The way to use the chatbot is not very complicated. |
| Social Influence  (SI) | SI 1 | If people around me are interested in chatbots, I would be interested. |
|  | SI 2 | People who help me cure TB will want me to use a chatbot. |
|  | SI 3 | If I say I use a chatbot, I see myself as a person who manages their own health. |
|  | SI 4 | If I use a chatbot, people around me will think I know a lot about tuberculosis. |
| Facilitating Conditions  (FC) | FC 1 | I can use the chatbot anytime, anywhere, using the internet. |
|  | FC 2 | I can be educated to use the chatbot anytime I want. |
|  | FC 3 | I have a smartphone or computer for chatbot. |
|  | FC 4 | I can get continuous guidance or help in using chatbot. |
| Attitude to Chatbot  (ATT) | ATT 1 | Using a chatbot sounds like a good idea. |
|  | ATT 2 | Using a chatbot will make the process of understanding tuberculosis more interesting. |
|  | ATT 3 | Chatbot seems to be fun. |
|  | ATT 4 | I like to use chatbot. |
| Behavioral Intention  (BI) | BI 1 | I'm very likely to use the chatbot soon. |
|  | BI 2 | I am willing to use a chatbot. |
|  | BI 3 | I plan to use a chatbot. |
|  | BI 4 | I want to use a chatbot. |
